# PCI-SS: MISO dynamic nonlinear protein secondary structure prediction

**DOI:** 10.1186/1471-2105-10-222

**Published:** 2009-07-17

**Authors:** James R Green, Michael J Korenberg, Mohammed O Aboul-Magd

**Affiliations:** 1 Department of Systems and Computer Engineering, Carleton University, Ottawa, Ontario, Canada; 2 Department of Electrical and Computer Engineering, Queen's University, Kingston, Ontario, Canada

## Abstract

**Background:**

Since the function of a protein is largely dictated by its three dimensional configuration, determining a protein's structure is of fundamental importance to biology. Here we report on a novel approach to determining the one dimensional secondary structure of proteins (distinguishing α-helices, β-strands, and non-regular structures) from primary sequence data which makes use of Parallel Cascade Identification (PCI), a powerful technique from the field of nonlinear system identification.

**Results:**

Using PSI-BLAST divergent evolutionary profiles as input data, dynamic nonlinear systems are built through a black-box approach to model the process of protein folding. Genetic algorithms (GAs) are applied in order to optimize the architectural parameters of the PCI models. The three-state prediction problem is broken down into a combination of three binary sub-problems and protein structure classifiers are built using 2 layers of PCI classifiers. Careful construction of the optimization, training, and test datasets ensures that no homology exists between any training and testing data. A detailed comparison between PCI and 9 contemporary methods is provided over a set of 125 new protein chains guaranteed to be dissimilar to all training data. Unlike other secondary structure prediction methods, here a web service is developed to provide both human- and machine-readable interfaces to PCI-based protein secondary structure prediction. This server, called PCI-SS, is available at . In addition to a dynamic PHP-generated web interface for humans, a Simple Object Access Protocol (SOAP) interface is added to permit invocation of the PCI-SS service remotely. This machine-readable interface facilitates incorporation of PCI-SS into multi-faceted systems biology analysis pipelines requiring protein secondary structure information, and greatly simplifies high-throughput analyses. XML is used to represent the input protein sequence data and also to encode the resulting structure prediction in a machine-readable format. To our knowledge, this represents the only publicly available SOAP-interface for a protein secondary structure prediction service with published WSDL interface definition.

**Conclusion:**

Relative to the 9 contemporary methods included in the comparison cascaded PCI classifiers perform well, however PCI finds greatest application as a consensus classifier. When PCI is used to combine a sequence-to-structure PCI-based classifier with the current leading ANN-based method, PSIPRED, the overall error rate (Q3) is maintained while the rate of occurrence of a particularly detrimental error is reduced by up to 25%. This improvement in BAD score, combined with the machine-readable SOAP web service interface makes PCI-SS particularly useful for inclusion in a tertiary structure prediction pipeline.

## Background

Proteins play critical roles in almost all biological activities within a living system. Since the function of a protein is largely dictated by its three dimensional configuration, determining a protein's structure is of fundamental importance to biology. Unfortunately, experimental methods for elucidating a protein's structure are often costly and are not always applicable [1]. Computational prediction techniques provide an attractive alternative; however, the accurate prediction of 3D protein structure directly from amino acid sequence data continues to elude researchers when homologous protein structures are not available (comparative modeling), or for longer domains (*de novo *modeling). As an intermediate but useful step, attempts have been made to determine the one dimensional secondary structure of proteins (distinguishing α-helices, β-strands, and non-regular structure) from primary sequence data [2]. A wide variety of methods have been applied to this problem including those based on artificial neural networks (ANNs) [3-8], hidden Markov models (HMMs) [8,9], information theory [5], linear programming [10], and linear discriminant analysis (LDA) [5], however no method has achieved the theoretical maximum predictive Q_3 _accuracy of 88% [2]. Interested readers are directed to an excellent review of the state of the art by Rost [2]. Note that this study focuses on predicting secondary structure of globular proteins. Excluded proteins include those with coiled-coil regions or trans-membrane domains.

Here we report on a novel approach to this problem which makes use of powerful techniques from the field of nonlinear system identification. Using divergent evolutionary profiles [11] as input data, parallel cascade identification [12] (PCI) is used to build multi-input single-output (MISO) dynamic nonlinear systems through a black-box approach in order to model the process of protein folding. The application of cascaded PCI classifiers suggested in ref. [13] is also used here to great advantage. While PCI proved to be a relatively accurate method of predicting secondary structure directly from sequence, PCI achieves its greatest accuracy when PCI-based classifiers are combined with PSIPRED [6], a leading ANN-based method, using a cascaded PCI classifier. When evaluated over a new test dataset of 125 protein chains sharing no significant sequence similarity to the training data for either method, this combination maintains the highest observed prediction accuracy while reducing the BAD score by up to 25%. The BAD score measures the percentage of misclassification errors confusing α-helices and β-strands which are known to be particularly damaging for inferring tertiary structure. PCI's ability to significantly reduce this error type while maintaining all other performance measures makes the PCI-PSIPRED combination particularly well suited for inclusion in tertiary structure prediction pipelines.

### PCI-SS Web Server

We have developed an advanced web server for PCI-based protein secondary structure prediction. This server, called PCI-SS, is available at . In addition to a dynamic PHP-generated web interface for humans, a Simple Object Access Protocol (SOAP) interface is added to permit invocation of the PCI-SS service remotely. Several other protein secondary structure human-readable web interfaces are currently available. While these interfaces work well for determining the structure of a single input sequence, such human-readable interfaces are not well suited to automated high-throughput analysis of multiple proteins. With the shift from the reductionist view, that seeks to analyse individual molecules in isolation, to the introduction of systems biology which examines the complex interactions of multiple molecules at a cellular or organism level, biologists are turning to high-throughput analyses that can characterize an entire proteome at once. Such analyses are often multi-faceted where, for example, protein structure, sub-cellular localization, interactions, and functional information are considered simultaneously to achieve more information than can be obtained through any single avenue of investigation. This requires that individual sources of information be combined into complex analysis pipelines. Again, human-readable web interfaces are not well suited for such pipelines since the input and output data is limited to unstructured text. Complex web agent scripts can sometimes be created to simulate the human interactions with a website and painstakingly parse the HTML output. However, such approaches, often referred to as 'scraping', are prone to failure when a service provider changes so much as the presentation format of the web site providing the service. A number of methods of secondary structure prediction are available for download, to be run locally such as PSIPRED [6] and Proteus, a highly accurate method which uses structural templates to augment secondary structure predictions [14]. However, a web service oriented architecture may be better suited to biologist users who do not want to download, compile, configure, and maintain software locally, including any required hardware.

Emerging web technologies such as Simple Object Access Protocol (SOAP) [15], WSDL [16], and XML [17] are useful for creating machine-readable interfaces to web services over HTTP. WSDL is used to define the method interface in a language-independent way. By separating the interface form the implementation, client programs can design for the fixed interface while service providers are free to manage the way in which the service is implemented. Furthermore, the WSDL interface enables rapid development of clients in many development languages (PERL, PHP, Java, C++, C#, etc). By encoding the input and output data using XML, complex data structures may be encoded in a self-describing way that simplifies automated method invocation and parsing of results. These are critical for the successful incorporation of a web service into a high-throughput analysis pipeline.

Although at least one other secondary structure makes use of SOAP for inter-process communication (e.g. PROSPECT-PSPP [18]), the interface is not made public nor is a WSDL interface definition provided. To our knowledge, this is the first publicly available SOAP interface for a protein secondary structure prediction method.

### Parallel Cascade Identification

PCI is a powerful method of nonlinear system identification that may be used to create a mimetic model of a dynamic nonlinear system given only knowledge of its input and output data [12,19]. No special statistical properties are required of the training data provided that they are sufficiently rich [12,19]. Note that any PCI model will have equivalent Volterra and Wiener expansions [12,19]. A PCI model consists of a parallel arrangement of cascade models where, in the present study, each cascade is composed of a dynamic linear (L) component followed by a static nonlinear (N) polynomial component. During training, cascades are added to the model sequentially, where each new cascade reduces the residual error remaining between the training output and the sum of the outputs of the previously added cascades. Prior to training a PCI model, four architectural parameters must be set in order to fix the model structure [12]. These are: the maximum lag, *R*, and anticipation length, *S*, of the dynamic linear component L; the degree, *I*, of polynomial used for the static nonlinearity N; and a constant, *P*, related to the minimum MSE reduction required of a candidate cascade before it is accepted into the parallel cascade model. In the present study, genetic algorithms (GAs) are used to sample this parameter space and to search for a suitable PCI architecture [20].

In order to use PCI to predict protein secondary structure, the problem must be recast into one of nonlinear system identification as follows: We seek to identify a MISO dynamic nonlinear system that can map sequence data onto secondary structure state data. It is conceivable that such a system exists since this mapping occurs *in vivo*. Prior to applying sequence data to the inputs of a PCI model, it must first be suitably encoded into a numeric matrix. In the present study, PSI-BLAST [11] is used to generate position-specific scoring matrices (PSSMs) from each query protein chain as first suggested by Jones [6]. For a query protein of length M, the corresponding PSSM is a matrix with M rows and 20 columns. In order to use PSSM data as input, we make use of a 20-input MISO PCI model, where each of the 20 inputs accepts one column of the PSSM. To classify new (i.e. query) sequences, the encoded input data is applied to the trained PCI model. The model output is then examined using a decision function in order to classify each input datum. A number of decision functions may be used to perform this final classification. In this study, the MSE-test [21,22] is used. The interested reader is referred to ref. [12] for a more complete treatment of the PCI algorithm, and to refs. [21-23] for a detailed discussion of the use of PCI to form bioinformatics binary classifiers.

## Results and Discussion

### Selection of sequence-unique training/testing data

Comparative modeling (or homology modeling) is by far the most accurate structure prediction method when a sequence-similar protein with known structure is available [2]. It follows that secondary structure prediction techniques are only needed when comparative modeling cannot be applied. Therefore predictions should be evaluated using a dataset where no homology exists between training and testing data. Gross overestimation of prediction accuracy may result when this is not ensured explicitly at the experiment design stage [2]. A list of 3107 sequence-unique protein chains was retrieved from the EVA system [24] on 2 May 2004. The dataset was then filtered resulting in 2713 chains (see Methods) and split into five subsets: The S1 subset was used as the optimization set. Subsets S2, S3 and S4 were used to train and test numerous models in a cross-validation scheme in order to explore a wide variety of algorithmic ideas. Once the algorithm exploration stage was complete, the "antiTest" dataset was formed from the union of S1, S2, S3, and S4 (2170 chains total). Penultimate classifiers were trained on the antiTest dataset and tested on the S5 test dataset. Note that subset S5 was reserved as a test set and was not used to train any classifiers (with the exception of the final evaluation over new EVA data as described below).

### Sequence-to-structure PCI-MSE classifiers

**Figure 1:**
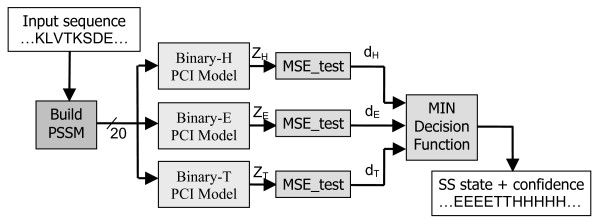
**Sequence-to-structure PCI classifier**. Creating a 3-ary sequence-to-structure PCI-MSE classifier from 3 binary PCI-MSE classifiers.

Three-state sequence-to-structure PCI classifiers were formed through a combination of three binary PCI classifiers as depicted in Figure 1 (see Methods section for details). Using the S1 optimization dataset (543 protein chains), genetic algorithms were applied to optimize the architectural parameters of each binary sequence-to-structure PCI model (see Methods). Table 1 shows the optimal parameters for each binary PCI model. The nonzero values for both *R *and *S *would seem to indicate that the secondary structure of the central residue is dependant on neighbouring amino acids both up- and downstream [25]. Following optimization of the PCI architectural parameters, sequence-to-structure PCI classifiers were trained over the antiTest dataset and were evaluated using the S5 test dataset. Matthew's correlation coefficients [26], Q_3 _accuracy, SOV score [27,28], and the BAD score [2] are reported in the first row of Table 2. Note that Q_3 _accuracy surpasses 73% and is approaching the state of the art for contemporary methods.

**Table 1:** Optimal PCI Architectural parameters

		**R**	**S**	**I**	**P**
	
	**Search space**	3 ≤ (*R *+ *S *≤ 29)		[1,8]	[5,50] (steps of 5)
**Sequence-to-structure**	**Binary_H**	10	9	3	35
	
	**Binary_E**	9	15	3	50
	
	**Binary_T**	5	7	2	40

**Structure-to-structure**	**Binary_H**	4	7	6	50
	
	**Binary_E**	4	1	7	45
	
	**Binary_T**	1	3	7	20

Optimal binary PCI architectural parameters following GA optimization over S1 dataset.

**Table 2:** PCI accuracy over S5 test dataset

	**CC_H_**	**CC_E_**	**CC_T_**	**Q_3_**	**BAD**	**SOV**
**Sequence-to-structure PCI classifier alone**	0.661	0.572	0.530	73.9%	2.72	61.8

**Cascaded PCI classifier**	0.693	0.595	0.547	75.5%	1.89	67.1

**Figure 2:**
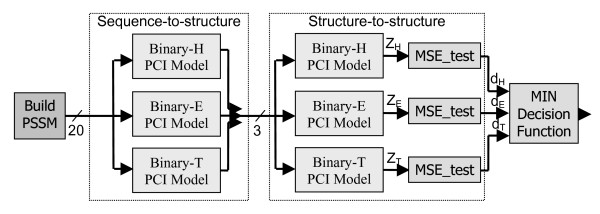
**Cascaded PCI classifier**. Cascaded PCI classifier formed from PCI sequence-to-structure models followed by a cascaded sequence-to-structure (post-PCI) classifier.

Sequence-to-structure (see Figure 1) and cascaded (see Figure 2) PCI classifier results over S5 test dataset of 543 chains.

### Cascaded PCI classifiers

Many contemporary secondary structure prediction methods have made use of cascaded classifiers [2]. The first classifier acts as a sequence-to-structure classifier (i.e. input data are sequence data and output data are structural states). The second classifier is a structure-to-structure classifier that examines a local window of predicted structure and hones the prediction of the structure state. These structure-to-structure classifiers capture purely structural relationships (e.g. α-helices must be at least 4 residues long to be stable) and correlations that may exist between adjacent structure states. Figure 2 illustrates a cascaded PCI classifier. Using the S1 optimization dataset, GAs were applied to optimize the architectural parameters (i.e. *R*, *S*, *I*, and *P*) of the structure-to-structure PCI classifier. Optimal architectural parameters are given in Table 1 for the structure-to-structure PCI models (henceforth referred to as post-PCI classifiers). Using those architectural parameters found to be optimal, a cascaded PCI classifier was trained on the antiTest dataset and evaluated over the S5 test dataset. Significant improvements in all measures of accuracy are observed in Table 2 when compared with the sequence-to-structure PCI-MSE classifier described above.

### Consensus combination of PCI with PSIPRED

**Figure 3:**
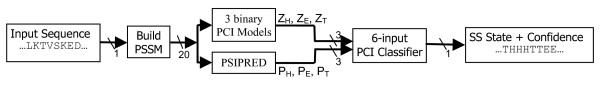
**PCI consensus classifier structure**. A 6-input cascaded PCI classifier is used to combine 3 outputs from binary PCI sequence-to-structure models with 3 distance outputs from PSIPRED [6].

The combination of multiple diverse predictors has been shown to improve secondary structure prediction accuracy when the individual predictors (i.e. experts) do not suffer from the same errors [2,5,29]. PCI has previously been successfully combined with hidden Markov models [21] and K-nearest-neighbour classifiers [22] for the analysis of proteins. Since PCI is a fundamentally different approach to secondary structure prediction from what is currently available, it may not generate the same prediction errors as other methods. A six-input post-PCI classifier was formed to combine the raw outputs of three binary sequence-to-structure PCI models with the three class distances produced by a PSIPRED [6] classifier as shown in figure 3. The post-PCI classifier was trained on the antiTest dataset, as were the sequence-to-structure binary PCI models used to generate the post-PCI input data. The prediction accuracy of PSIPRED alone and in combination with PCI classifiers over the S5 test dataset are given below in Table 3. It is clear from the results below that PCI is augmenting the PSIPRED predictions when they are combined using post-PCI. Not only are fewer errors committed, but the BAD score is reduced by 25%. This may indicate that the structure predicted by the combined classifiers could be more useful to subsequent tertiary structure prediction methods than PSIPRED predictions alone [2].

**Table 3:** Combination of PCI with PSIPRED

	**CC_H_**	**CC_E_**	**CC_T_**	**Q_3_**	**BAD**	**SOV**
**PSIPRED alone (no post-PCI)**	0.727	0.646	0.585	77.8%	1.49	68.9

**Post-PCI(PCI & PSIPRED)**	0.740	0.647	0.592	78.5%	1.12	69.8

Prediction accuracy over the S5 test dataset (543 chains) for PSIPRED alone and for the post-PCI combination of PSIPRED with 3 binary PCI model outputs.

### Final EVA test set

In order to compare PCI-based classifiers with contemporary classifiers based on different approaches, a common dataset of 125 protein chains that were guaranteed to be non-homologous to all protein data used to train all eight methods was extracted from the EVA system [24] (see Methods for details). This dataset provides a unique opportunity to directly compare PCI's performance with eight leading methods in a fair and objective way. The benefit of using results from the EVA system is that we are guaranteed that all test proteins are dissimilar to all training proteins for all methods. No such guarantee is available if non-EVA methods are applied to the test dataset now, since there is no way to ensure that these same proteins were not used in the training of each method. We therefore restrict our comparison to methods evaluated by the EVA system. Excluded methods of interest include one based on support vector machines which appears to achieve similar prediction accuracy to PSIPRED [30], Proteus [14], and Yaspin [8], which uses a hidden Markov model to refine the 7-state predictions from a neural network. Although Yaspin was included in EVA for a short time, unfortunately, archived EVA results were only available for a small number of the 125 test proteins and therefore this method was excluded. Results over those proteins for which archived Yaspin were available showed performance on par with cascaded PCI.

A cascaded post-PCI classifier (as shown in Figure 2) was trained over the entire original dataset of 2713 protein chains, as was a post-PCI combination of 3 binary PCI model raw outputs with three PSIPRED distance outputs. Archived results over the 125 protein chains comprising the final EVA test set were extracted from the EVA system [24] for 9 methods (see Methods). Columns 2–4 of Table 4 show the prediction accuracy when all 5627 residues from the 125 chains are pooled together, while the last four columns report the average performance when results are calculated on a per-chain basis as is done in EVA [24]. Note that scores computed using this latter approach are typically lower than the first approach due to the effect of poor classifier performance over shorter protein chains. As can be seen in Table 4, the cascaded post-PCI classifier ranks reasonably well among the top contemporary methods, with higher SOV and Q3 scores than 4 of 9 methods. Furthermore, the post-PCI combination of PSIPRED-local with 3 binary PCI outputs achieves the highest Q3 and SOV scores observed in Table 4, and also displays the lowest BAD score of any method. While the increase in Q3 and SOV are minimal when compared to PSIPRED alone, the decrease in BAD score (up to 25%) is statistically significant with a p-value of 0.004 when a paired t-test is applied to BAD scores measured over each of the 125 proteins. This significant reduction in the rate of occurrence of helix-strand confusion is expected to make this method significantly better suited to inclusion in tertiary structure prediction pipelines.

**Table 4:** Results over the final EVA test

	**Avg per residue**			**Avg per chain**			
	
**Method**	**CC**	**Q_3_**	**BAD**	**CC**	**Q_3_**	**BAD**	**SOV**
**PHD**	0.619	76.09	2.65	0.631	75.96	2.82	71.4
	
**PHDpsi**	0.619	76.09	2.65	0.631	75.96	2.82	71.4
	
**PROF_king**	0.577	72.65	3.70	0.594	72.86	3.38	66.8
	
**PROFsec**	0.651	77.72	2.36	0.659	77.70	2.49	75.3
	
**PSIPRED-live (UniREF100 DB)**	0.668	79.02	2.01	**0.675**	78.86	2.12	76.1
	
**Sable**	0.633	76.74	2.62	0.634	76.50	2.74	73.9
	
**Sable2**	0.651	77.95	1.86	0.644	77.45	2.05	73.0
	
**SCRATCH (SSPro3)**	0.616	76.07	3.24	0.622	76.15	3.20	70.6
	
**SSPro4**	0.643	77.69	2.43	0.642	77.58	2.46	72.0

**Cascaded PCI**	0.632	76.45	2.70	0.624	76.31	2.69	72.0
	
**PSIPRED-local (frozen nr DB)**	0.676	79.44	2.13	0.658	79.36	2.20	75.5
	
**post-PCI(PCI &PSIPRED)**	**0.682**	**79.58**	**1.60**	0.656	**79.37**	**1.68**	**76.4**

**Bad-Score-Rule**	0.679	**79.59**	1.98	0.659	**79.45**	2.10	75.7

Results over the final EVA test set of 125 new protein chains dissimilar to all training data. CC denotes the average Matthews' correlation coefficient observed for the three classes. 'Avg per residue' results are calculated over the pool of all residues in the dataset whereas 'Avg per chain' results are compiled for each chain prior to computing the average. PSIPRED-local refers to the output of PSIPRED v2.45 run locally when provided with PSSM data generated from the filtered NCBI non-redundant nr database as frozen on 3 May 2004. PSIPRED-live indicates the performance of the actual PSIPRED server [31] as of the day that each protein chain was added to the EVA system. Bad-Score-Rule in the last row shows the results when a rule is applied to combine postPCI with PSIPRED to optimize Q3 score at the cost of BAD score (see text for details).

Note that it is possible to improve the Q3 score slightly (increase from 79.37 to 79.45; the highest observed Q3 score) at the expense of performance on the BAD score (increase 1.68 to 2.10; although still lower than PSIPRED alone). This may be accomplished by accepting the postPCI classification whenever PSIPRED and the postPCI consensus classifier differ on a helix-strand prediction. This so called 'BAD score rule' replaces the PSIPRED prediction with the postPCI consensus prediction whenever PSIPRED predicts strand and postPCI predicts helix, or when PSIPRED predicts helix and postPCI predicts strand. When this rule is applied to the 125 proteins in the EVA dataset, 138 replacements are made during structure prediction. These results are shown in the last row of Table 4.

### Quality of training data

When developing PSIPRED, Jones was careful to select structures that were of high quality, accepting only training protein chains whose structure had been determined through X-ray crystallography with a RMS of less than 1.8 angstroms [6]. When constructing the list of unique proteins, the EVA system aims to select the chain of highest quality from within a family of similar chains. However, no minimum structure quality criterion is imposed for inclusion in the list [24]. To gauge the importance of using only high quality precisely defined structures as training data, the PISCES system [31] was used to filter the antiTest dataset of training protein chains. Only those chains that were solved via X-ray crystallography and whose structures were known within 2 angstroms were retained. This reduced the list of training chains from 2170 to only 620. A PCI-MSE classifier was trained on the filtered antiTest dataset and then evaluated on the unfiltered S5 test dataset. A full 1% decrease in Q_3 _accuracy was observed for the PISCES-trained PCI-MSE classifier compared to the classifier trained over the unfiltered dataset. This is likely due to the reduced number of training data provided by the filtered training dataset. PCI therefore appears to be relatively robust to the quality of the training data.

## Conclusion

In the present study, PCI was used to form the basis for a number of protein secondary structure classifiers. The use of PSI-BLAST [11] to iteratively create multiple sequence alignments of distantly, but significantly related proteins and to build PSSMs was critical to the success of this study. PCI-based classifiers that used PSSM input data were shown to be effective on an independent dataset of new protein chains, and compared favourably with 9 leading contemporary structure prediction methods. The use of cascaded structure-to-structure PCI-MSE classifiers (post-PCI) appears to be a highly effective method to refine sequence-to-structure PCI-MSE classifier outputs and also to combine PCI with other methods. When post-PCI is used to combine a sequence-to-structure PCI-based classifier with a PSIPRED [6] classifier, overall performance is maintained while significantly (p < 0.01) reducing the rate of occurrence of a particularly detrimental error by up to 25%. In fact, these post-PCI classifiers achieve the highest Q_3 _and SOV accuracies and lowest BAD score observed for any method evaluated in the present study over a novel set of 125 protein chains guaranteed to be dissimilar to all proteins used to train all methods.

The use of structure-to-structure consensus PCI classifiers to combine sequence-to-structure PCI classifiers with a leading ANN-based method [6] to achieve an overall increase in quality and accuracy of predicting secondary structure is an exciting outcome. It may be possible to use post-PCI to combine PCI-based classifiers with other types of classifiers, including HMM-based classifiers. While predicting secondary structure is a useful intermediate step, the ultimate goal of protein structure prediction is to predict the complete 3D structure of the active conformation(s) of a protein given knowledge only of its sequence and its environment. The application of PCI to tertiary structure prediction is the logical next step towards this goal. Gaining a better understanding of the many post-translational modifications that may occur to a protein that can significantly alter a protein's function is also a worthy goal and PCI has already shown promises in this area as well [32].

**Figure 4:**
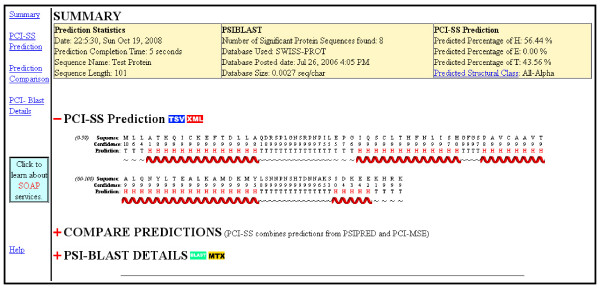
**PCI-SS webserver screen capture**. Screen capture of human-readable PCI-SS webserver results page. Users can download predicted structure in tab-separated value or XML format. Users can also view a comparison of the consensus prediction against the component classifiers, and can download PSI-BLAST search results and PSSM data.

A web server called PCI-SS was created with both a human-readable and machine-readable interface. The human interface follows the design of many existing systems, but adds the ability to download the structure prediction in XML format for easy parsing. Intermediate results are also provided, including PSI-BLAST PSSM data and a comparison between the consensus PCI-SS prediction and its constituent methods. A sample screen shot is provided in figure 4. The machine-invocable SOAP interface is unique in that it allows the language-independent creation of custom application-specific programs which can invoke PCI-SS remotely while completely bypassing the human-readable web interface. This ability combined with XML encoding of the resulting prediction makes PCI-SS particularly well suited to incorporation into custom multi-faceted protein analysis pipelines. To facilitate development of custom user programs, two sample PERL scripts are provided on the website as examples of how to remotely invoke the method and parse the results. The SOAP webserver and the significant decrease in BAD score will make PCI-SS of particular interest to developers of tertiary structure prediction methods.

## Methods

### Parallel Cascade Identification

PCI is a powerful system identification technique that aims to build a mimetic dynamic nonlinear model given the input and output data from a real system. Interested readers are directed to ref [12] for a detailed description of the algorithm. A PCI model consists of a parallel arrangement of cascade models. Training the i^th ^cascade path begins with fitting the (*R*+*S*+1) taps of the FIR filter in the L component. These values are taken from cross-correlations between the input data, x [n], and the desired cascade output, y_i _[n], computed for offsets in the range [-*S*, +*R*]. If a higher order cross-correlation is computed, then a one-dimensional slice is used in combination with strategically placed Kronecker delta functions [12]. The output of the i^th ^L component, u_i _[n], is computed via a convolution of the FIR filter and the input data. To overcome transient effects introduced by the convolution of the FIR filters with a protein chain input, each protein chain was first zero-padded by *R *residues at the start of each chain, and by *S *residues at the end of each chain, after each cross-correlation (to obtain the FIR filter) was computed [25]. The polynomial of order *I *forming the i^th ^N component is then used to best-fit u_i _[n] to y_i _[n]. The residual, y_i+1 _[n], between the actual cascade output, z_i _[n], and the desired cascade output, y_i _[n], forms the desired output for training the next cascade path. For the case of a multi-input single-output (MISO) PCI model, as is used throughout this study, the cross-correlations to fit the FIR filter are computed between the desired cascade output and one or more inputs selected randomly with replacement [12].

Before accepting a new candidate cascade into the PCI model, a minimum mean squared error (MSE) reduction criterion may be imposed. The stringency of this test is controlled by the architectural parameter *P *and is related to a standard correlation test to help ensure that the model will not fit solely noise [12]. Training continues with the fitting of new cascade paths until either 1) a pre-determined maximum number of candidate cascades are consecutively rejected (a value of 150 is used in the present study), or 2) a maximum number of cascades, *maxC*, are identified and added to the model. During optimization, the value of *maxC *was set to 85, primarily to reduce the computational requirements of assessing each PCI parameter set. The value of *maxC *was increased to 500, without risk of overfitting, when training the final PCI classifiers over the antiTest dataset since the size of the training data increased in length to 449112 residues as compared to only 112685 residues for the optimization subset. Note that *maxC *is an *upper limit*, and that the actual number of cascades in a model may be much less due to the use of the MSE reduction criterion. In fact, the number of cascades in the final PCI-MSE sequence-to-structure binary models were E = 482, T = 295, H = 500 while the post-PCI consensus classifier which combined PCI and PSIPRED had fewer cascades (E = 205, T = 250, H = 447).

Secondary structure prediction is a 3-state problem. By using a multi-level output (e.g. H = 1, T = 0, E = -1), a single 3-state PCI classifier can be used to achieve this. Rather than using a single 3-state PCI classifier, we can instead create three binary sub-problems, as depicted in Figure 1. Here, each binary model is trained on a specialized version of the training data where the output has been set to 1 for one primary secondary structure state, and -1 for the other two states. For example, when training a binary_H classifier, the output data for the primary state, H, were mapped to 1, while the remaining states, E and T, were mapped to -1 [33]. Each binary classifier is therefore an expert in recognizing one of the three states. During testing, the MSE-test score from each binary classifier is computed. The MSE-test score is a measure of mean-squared error between the actual model output and the nominal model output for the class of interest, scaled by the variance observed during training. More details are provided in ref [21,22]. A 3-input MIN decision function (see Figure 1) examines the three MSE-test scores treating them as distances: the state for which the MSE score is smallest is selected. A measure of confidence is calculated as follows:



where *d*_1 _and *d*_2 _are respectively the smallest and next-smallest MSE-test scores from all three binary classifiers.

### Preparation of the datasets

A list of 3107 sequence-unique protein chains was downloaded from the EVA system on 2 May 2004. Proteins whose amino acid sequence was not known with certainty or whose secondary structure was not available were removed from the dataset. This filtering resulted in 2713 protein chains remaining, of which the average chain length was 204 residues, the minimum and maximum chain lengths were 11 and 1290 residues respectively, and the total number of amino acid residues was 554085. The dataset was then divided into 5 subsets: S1 (543 chains), S2 (543 chains), S3 (542 chains), S4 (542 chains), and S5 (543 chains). Position-specific scoring matrices (PSSM) were computed for each sequence using PSI-BLAST [11] run for 3 iterations with a E-score threshold of 0.001 as used by PSIPRED [6].

Due to the nature of the EVA system, any proteins added to the system after the date on which a protein chain list is downloaded from EVA are guaranteed not to be homologous to any proteins contained on that chain list [24]. As just stated, such a protein chain list was downloaded on 2 May 2004 and those data were used to develop the PCI-based classifiers described in this study. On 5 April 2007, at the end of the study, a list of 365 new protein chains added to the EVA system since 2 May 2004 was downloaded and was used to construct a final test dataset. Of the 365 newly added protein chains, a subset of 125 protein chains had been tested by EVA on their dates of deposition into the PDB [34] against a battery of 9 contemporary methods. Unfortunately, EVA had not run all 365 new proteins against all 9 methods. The subset of 125 protein chains was selected to form the final EVA test set since archived results were available from EVA for each chain for all 9 methods. This dataset provides a unique opportunity to directly compare PCI's performance with a number of leading methods in a fair and objective way. The final EVA test set totalled 12905 residues, with an average of 103 residues per chain, and exhibited minimum and maximum chain lengths of 30 and 644 residues respectively. Note that a number of protein chains had one or more unknown residues in their sequence. These chains were kept in the test set since PSI-BLAST is able to handle such residues and still produce meaningful PSSM data.

The final PCI-based classifiers and post-PCI consensus combinations of PCI and PSIPRED were trained on the complete dataset of all original EVA data downloaded in May of 2004 (i.e. subsets S1 through S5) for a total of 2713 protein chains. These methods were then evaluated using the final EVA test dataset which shared no significant sequence similarity with any of the 2713 training proteins. Detailed prediction results were downloaded and parsed from the EVA system for 9 contemporary methods over the same dataset of 125 protein chains. The following methods are included in the comparison: PHD [3], PHDpsi [4], PROF_king [5], PROFsec [3], PSIPRED [6], Sable [10], Sable2 [10], SCRATCH (SSPro3) [7], and SSPro4 [7] and represent methods using ANNs [3-7,10], information theory [5], and LDA [5]. Note that it would appear that the EVA system is no longer being updated on a regular basis however it remains a unique resource of results over multiple methods for a large database of sequence-dissimilar proteins.

PSI-BLAST requires a database of protein sequences to search against. In this study, a local copy of the NCBI "nr" (non-redundant) database was made on 21 June 2004, containing 1,865,463 sequences totalling 619,299,334 residues. Prior to use, this database was filtered for unwanted low complexity or coiled-coil elements using the pfilt program written by David Jones as part of the PSIPRED (v.2.45) suite of programs [6]. Note that the SwissProt database is used on the live webserver version to reduce computational time. No significant or systematic change in performance is observed when the sequence database is changed.

Ground truth secondary structure assignments for each protein chain were obtained using the DSSP program [35]. In the current study, the eight DSSP output classes are mapped to three states as follows: H={H,G,I}, E={E,B}, T={T,S,-}, where '-' denotes 'other'. This conversion is recommended as being a conservative approach [2]. This resulted in 20% of residues assigned to class E (β-strands), 33% of residues assigned to class H (α-helices), and the remaining 47% of residues assigned to class T (loops, turns, or non-regular structure).

### Measuring prediction accuracy

Prediction accuracy is often measured using the Q_3 _score which is defined as the percentage of all residues that were predicted to be in the correct secondary structure state. By using a correlation coefficient [26], a more relevant evaluation of prediction accuracy is achieved. Matthews' correlation coefficient [26] (CC) combines sensitivity and specificity into a single measure and is widely employed to measure prediction accuracy. One weakness of the Q_3 _score is that it considers all errors to have equal cost despite the fact that not all types of errors are equally detrimental to the usefulness of a secondary structure prediction [27,28]. The output of secondary structure prediction systems are often used to guide methods of tertiary structure prediction. Errors that involve the misclassification of a strand as a helix, or vice-versa, are particularly damaging to the eventual accuracy of the tertiary structure prediction [2]. To reflect this fact, it is common to report not only the Q_3 _and Matthews' correlation coefficient, but also the BAD score [2] for each secondary structure prediction. The BAD score is defined as the percentage of all predictions in which a strand and a helix state were confused. Lastly, we also report the segment overlap (SOV) score reflecting the degree of overlap between predicted and observed structural segments as defined in ref. [27,28].

### Optimization of PCI parameters through genetic algorithms

Each potential parameter set (consisting of *R*, *S*, *I*, and *P*) was represented by a chromosome having four genes. These were: 1) total memory length (not counting lag 0), *g*_1 _= (*R*+*S*); 2) degree of anticipation, *g*_2 _= (*S*/(*R*+*S*)); 3) degree of polynomial, *g*_3 _= *I*; and 4) cascade acceptance criteria, *g*_4 _= *P*. Possible values of the *g*_4 _gene were limited to multiples of 5 since a more coarse-grained search over a wider range was found to be most suitable for the *P *parameter. In order to evaluate parameter sets, 3-fold cross-validation testing was performed over the S1 optimization subset of 543 protein chains (112685 residues total). The average Matthews' correlation coefficient observed over the three folds was used as the criterion function. The GA was run for 26 generations with a population size of 24 chromosomes. The mutation rate was set to 0.25 and Booker's variable cross-over rate was used [36]. Although the parameter set which gave the highest prediction accuracy over the optimization data set was ultimately selected, several parameter sets were identified which gave suitable prediction accuracy over the cross-validation subsets and PCI's accuracy was not highly sensitive to architectural parameter values.

The following method was used in order to optimize the architectural parameters of three binary 3-input structure-to-structure PCI-MSE classifiers (or post-PCI classifiers) depicted in Figure 2: Three binary sequence-to-structure PCI models, characterized by the parameters given in Table 1, were trained on the S1 subset and then applied to the S1 subset thereby providing three raw PCI output signals. During optimization of the structure-to-structure binary PCI models, these three raw output signals were applied to the three inputs of each binary post-PCI model.

### Consensus combination of PCI with PSIPRED

In this study, PCI was used to build a consensus classifier which combined PCI-MSE and PSIPRED [6], a leading ANN-based prediction method [24], as shown in figure 3. A pre-trained copy of PSIPRED v2.45 was downloaded, compiled, and run locally such that the source of the PSSM data could be controlled. The online "live" version of PSIPRED (found at ) makes use of a slightly different sequence database for generating the PSSM data than is used in this study, and its sequence database is also updated weekly [37]. When run locally, the PSIPRED program provides three output values for each residue, indicating the likelihood that each residue belongs to each of the three secondary structure classes. Each likelihood value fell in the range [0,1] and was transformed into a distance by subtracting each likelihood value from 1. A number of approaches to combining PCI and PSIPRED outputs were evaluated over the cross-validation subsets (S2, S3, and S4). The use of a 6-input PCI consensus classifier (i.e. a structure-to-structure cascaded PCI-MSE classifier) was identified as the most promising approach where the three PSIPRED distances were combined with three binary PCI model outputs. The so-called post-PCI classifier was characterized by the structure-to-structure PCI architectural parameters listed in Table 1 (i.e. optimization was not repeated). Note that a single 6-input 3-state post-PCI classifier may have been used in place of 3 binary 6-input PCI classifiers, but early testing on the cross-validation subsets showed inferior performance with this approach.

## Authors' contributions

JRG developed the prediction software, assembled the data sets, carried out the analysis, and drafted the manuscript. MOA developed the PCI-SS web server, assembled the final EVA test dataset, and ran the final comparison between PCI-SS and the other 9 methods. MJK conceived of the study, participated in the design of the study, contributed to algorithm development, and helped to draft the manuscript. All authors read and approved the final manuscript.
